# Activity of Daily Living and Depressive Symptoms in Chinese Older Adults: A Latent Profile and Mediation Analysis

**DOI:** 10.3389/ijph.2025.1608149

**Published:** 2025-05-30

**Authors:** Peng Chen, Wenjian Xu

**Affiliations:** Department of Sociology and Psychology, School of Public Administration, Sichuan University, Chengdu, Sichuan, China

**Keywords:** ADL, depressive symptoms, life satisfaction, latent profile analysis, Chinese older adults

## Abstract

**Objectives:**

This study aims to examine vulnerable ADL-based subgroups of Chinese older adults, their links to depressive symptoms, and life satisfaction as a mediating factor.

**Methods:**

We screened 8,211 participants aged 60 years and above who met the inclusion criteria from 2018 CHARLS. The different subgroups of ADL were identified by latent profile analysis. Life satisfaction and depressive symptoms were compared among the various ADL subgroups. Mediation analysis helped investigate the mediating role of life satisfaction between the various subgroups of ADL and depressive symptoms.

**Results:**

Two vulnerable subgroups of ADL were identified (*Low Damaged* class and *High Damaged* class), along with another subgroup of ADL (*Not Damaged* class), comprising the majority of Chinese older adults. The vulnerable subgroups of ADL had significantly lower life satisfaction and higher levels of depressive symptoms. The relationship between depressive symptoms and the vulnerable subgroups of ADL was partially mediated by life satisfaction.

**Conclusion:**

The results emphasize the role of life satisfaction in linking ADL with depressive symptoms, indicating potential areas for interventions to reduce depressive symptoms among older adults. This study is limited by its cross-sectional design precluding causal inference, reliance on self-reported data and unexplored moderating factors.

## Introduction

Worldwide, countries are experiencing a gradual aging of their populations, which is expected to significantly impact the economy, society, and healthcare systems [1]. It is anticipated that individuals aged 60 years and older will rise from 841 million in 2013 to more than 2 billion in 2050, accounting for 21.1% of the world’s population [2]. Official data show that China has the highest older adult population, with 297 million people over 60 years of age, accounting for over 20.0% of the total population [3, 4]. By 2035, more than 400 million people will be 60 years of age or older in China, making up more than 30.0% of the country’s total population [5]. With this continuous increase, their potential health problems have garnered significant social attention [6, 7].

The mental health of older adults is receiving more attention as the population aging trend continues, particularly with regard to depressive symptoms. A meta-analysis revealed that over one-third of the global older adults population suffers from depressive symptoms [8]. In China, a study involving 752 older adults indicated that 5.3% have minor depressive disorders, 4.8% have dysthymia, and 10.2% have major depression [9]. Studies have found significant differences in the occurrence of depression among older adults across various socio-demographic variables, such as gender, age, urban-rural residence, and marital status [9–11]. Additionally, physical health factors and depressive symptoms are significantly associated [11–13]. Therefore, focusing on the key factors influencing depressive symptoms in the elderly and examining the underlying mechanisms is necessary.

### Activities of Daily Living

Generally, as people age, they enter the senile stage and their physical functions decline, leading to limitations in performing activities of daily living (ADL) [14, 15]. ADL allows for maintaining independence in daily life, and is generally divided into basic activities of daily living (BADL; such as bathing, clothing, and eating) and instrumental activities of daily living (IADL; such as cleaning, shopping, and preparing meals) [10, 16]. ADL is typically used to measure physical functional limitations, which is an important indicator of the degree of impairment in the older adults [6]. An investigation of Chinese older adults revealed that the total rate of functional impairment was as high as 41.0%, with age-specific percentages for respondents aged 65–79, 80–89, and 90–99 reaching 6.9%, 23.6%, and 42.7%, respectively [17]. A study from Europe showed that older people were increasingly likely to transition to disability and dependency before the age of 70 [18]. A study on older East Asian adults living in the United States showed that increasing age was associated with a significant probability of transitioning to disability and experiencing reduced mobility [19]. Therefore, the decline in ADL levels with age is specially significant for older adults.

There are significant differences in the ADL of older adults across all dimensions [20, 21]. Traditional research methods for classifying ADL severity often rely on a paradigm that simply aggregates variables across dimensions, potentially overlooking heterogeneity within categories [13, 22, 23]. Individuals within the same category might exhibit diverse characteristics [21]. Further, there is a limited understanding of the living conditions and actual needs of the older adults [24]. By contrast, latent profile analysis (LPA) can identify and describe this within-category heterogeneity [25]. This method can effectively reflect various potential characteristics or patterns of ADL and identify distressed groups among the older adults [26]. Moreover, tailored and precise intervention programs can be provided to older adults, with varying levels of ADL, and the functional status and risk factors of older adults can be elucidated for clinicians and caregivers. This can facilitate a more targeted diagnosis, treatment, nursing, and rehabilitation services for older adults [20, 24]. Therefore, this study aimed to examine the scientific subgroups of ADL in older adults using a nationally-representative sample.

### Activities of Daily Living and Depressive Symptoms

Depressive symptoms are particularly prevalent among older people, being characterized by low mood and discomfort [27]. Owing to significant changes in bodily state, residence, and social position, depressive symptoms are comparatively common among older adults, considerably affecting their daily lives and health quality [10, 28]. Researchers have extensively investigated risk factors for depressive symptoms, among which ADL features prominently [10, 12, 13]. According to the diathesis-stress model, stress significantly affects an individual’s mental health and can originate from acute events, such as sudden traumatic incidents, and/or ongoing conditions such as disabilities and chronic illnesses [29, 30]. Thus, a decline in ADL levels is regarded as a chronic stressor, and can influence depressive symptoms in older adults [31]. Cognitive changes or decline, disruptions of daily activities, negative self-concepts, and attribution patterns are important manifestations of daily stress in older adults [32–34]. First, the level of ADL decreases with age, indicating a change in cognitive performance, and previous studies have shown a significant correlation between cognitive impairment and depressive symptoms [33, 35, 36]. Second, reduced mobility can lower the frequency of performing leisure activities for older persons, which has long been seen as an important means of preserving mental health [34]. In addition, older adults with limited activity may have few social activities, lack interpersonal communication, and be more prone to depressive symptoms [34]. Finally, the most immediate pain caused by death is separation and loss, and the inability to cope with loss is considered the primary driving factor of depressive symptoms [32]. Thus, older adults with low levels of ADL often experience negative thoughts and a sense of self-negation when facing death, which can trigger various negative emotions, particularly depressive symptoms [32]. Furthermore, a longitudinal study has revealed that older adults with low, moderate, or severe disabilities exhibit significantly higher rates of cognitive impairment than those without physical disabilities [37]. Thus, there are possibly significant variations in depressive symptoms among older adults with varying levels of impairment [33].

Therefore, using ADL to identify particular risk categories of older persons with poor physical function is crucial. This will provide both a deeper comprehension of how this factor affects depressive symptoms and effective intervention and prevention strategies for specific risk groups among older adults [20, 26]. However, no study has explored the relationship between the specific risk subgroups of ADL and depressive symptoms in Chinese older adults. Therefore, this study sought to examine how ADL subgroups differ in terms of life satisfaction and depressive symptoms.

### The Role of Life Satisfaction as a Potential Mediator

Life satisfaction is typically used to subjectively evaluate the quality of life for people, referring to the degree of satisfaction derived from the fulfillment of their needs and desires [38]. According to the diathesis-stress model, the absence of positive factors can increase susceptibility to psychological disorders, and life satisfaction is an important protective factor for enhancing positive psychological resources [39, 40]. Additionally, enhancing life satisfaction positively affects individuals’ health, with cross-cultural consistency [41–44]. Moreover, depressive symptoms are important indicators of mental health, with previous research demonstrating that life satisfaction can significantly impact depressive symptoms in older adults [45]. From a stress perspective, life satisfaction can influence specific psychological resources in older adults, thereby alleviating or safeguarding mental health and decreasing depressive symptoms [46, 47].

ADL is a significant risk factor for older adults’ life satisfaction [48]. Previous research has indicated that physical dysfunction can limit the participation of older adults in social activities, decrease social engagement and support, and ultimately reduce their life satisfaction [49]. Meanwhile, providing care and nursing for disabled older adults may impose a significant burden on the family [50]. The inability to perform daily tasks independently forces older adults to depend on others, eroding their autonomy and self-control. This situation can lead to feeling hopelessness and reduce life satisfaction [50]. Therefore, the association between ADL and depressed symptoms may be mediated by life satisfaction. However, a previous study has found that people with mobility disabilities may construct life satisfaction differently from those without disabilities [51]. Consequently, for older adults, there may be variations in the impact of ADL on depressive symptoms with different levels of physical function, as mediated by life satisfaction. Therefore, identifying the potential risk subgroups of ADL in older adults through LPA is both feasible and important. It can help explore the underlying mechanisms and provide effective support and intervention for vulnerable older adult subgroups [26, 52]. Thus, this study also investigates the mediating role that life satisfaction plays in the association between different subgroups of ADL and depressive symptoms.

## Methods

### Sample

The CHARLS is a representative longitudinal survey in China. It has been widely used in previous studies [53, 54]. The data for this study is publicly accessible CHARLS data collected in 2018 (wave 4), comprising 17,708 participants [55]. Based on the purpose of this study, we formulated the inclusion criteria for the research subjects as follows: aged 60 and above; socio-demographic information including gender, education level, marital status, and household registration; 6-item BADL Scale and 6-item IADL Scale; 10-item Center for Epidemiological Studies Depression Scale (CES-D-10); single-item score from the Satisfaction with Life Scale. Based on these inclusion criteria, we selected 8,211 subjects from the CHARLS database.

### Instruments and Measures

#### Activities of Daily Living

The 6-item BADL Scale and 6-item IADL Scale in the CHARLS data were used to assess ADL. The BADL Scale contained six questions, including dressing, bathing, eating, getting in/out bed, using the toilet, and controlling urination or defecation. The IADL Scale contained six questions, including doing housework, meal preparation, shopping, phoning, taking medication, and managing money. The answers included four options ranging from 1 (no difficulty) to 4 (cannot do it). We added the scores for each item. The total score ranged from 12 to 48, and a higher score indicated a higher level of physical functional limitation [10]. Previous study indicated that these two scales had sufficient validity and reliability [56]. The Cronbach’s Alpha values for this sample were 0.76 for the BADL Scale and 0.83 for the IADL Scale.

#### Depressive Symptoms

The CES-D-10 in the CHARLS data was utilized to evaluate depressive symptoms [57]. It contained ten questions, such as I felt “fearful,” “everything I did was an effort,” and “depressed.” The answers included four options from 1 (rarely or never) to 4 (always). We added the scores for each item. The total score was ranged from 10 to 40, and a higher score indicated a higher level of depressive symptoms [58]. Previous study indicated that this scale had sufficient validity and reliability [54]. The Cronbach’s Alpha value was 0.81.

#### Life Satisfaction

Life satisfaction was measured using one of the items from the Satisfaction with Life Scale in the CHARLS data [59], “How satisfied are you with your life.” There were five options from 1 (fully satisfied) to 5 (not at all satisfied). A higher score indicated a lower level of life satisfaction. This single item has been found to perform as validly as the whole Satisfaction with Life Scale [60].

#### Covariates

For older adults, previous research has highlighted the significant impact of socio-demographics on depressive symptoms, including gender, age, education level, marital status, and household registration [9–11]. Therefore, the following socio-demographic factors were utilized in the analysis as covariates: gender (women vs. men), age (in years), education level (junior high school and below vs. high school and above), marital status (divorced or widowed vs. married or cohabited), and household registration (non-agricultural account vs. agricultural account).

### Analytic Strategies

First, using SPSS 22.0, descriptive statistics were conducted. Next, to identify potential subgroups with different ADL types, LPA was implemented with the 6-item BADL Scale and 6-item IADL Scale in Mplus 7.4 [61]. By employing probabilistic models, LPA determines the probability of each individual belonging to different categories, grouping individuals with similar response patterns into the same latent class [25]. This approach minimizes within-class differences and maximizes between-class differences, resulting in more refined classification outcomes [25]. Additionally, existing research has demonstrated that traditional methods have certain limitations in explaining data heterogeneity, whereas LPA shows significant advantages in handling complex heterogeneous data, yielding more scientifically valid classification outcomes [62]. We started with a one-class model and kept on until fit indices were not able to be improved much. We assessed the models based on the fitting indexes of AIC, BIC, aBIC and, entropy value. Additionally, LMR and BLRT were performed to compare the potential profile models. Moreover, using SPSS 22.0, analysis of variance was used to compare life satisfaction and depressive symptoms among different subgroups of ADL [63]. Building on previous research practices, after incorporating all selected covariates into the model simultaneously, the mediation effect of life satisfaction between subgroups of ADL and depressive symptoms was examined using relative mediation analysis [64, 65].

## Results

### Latent Classification of ADL

**FIGURE 1:**
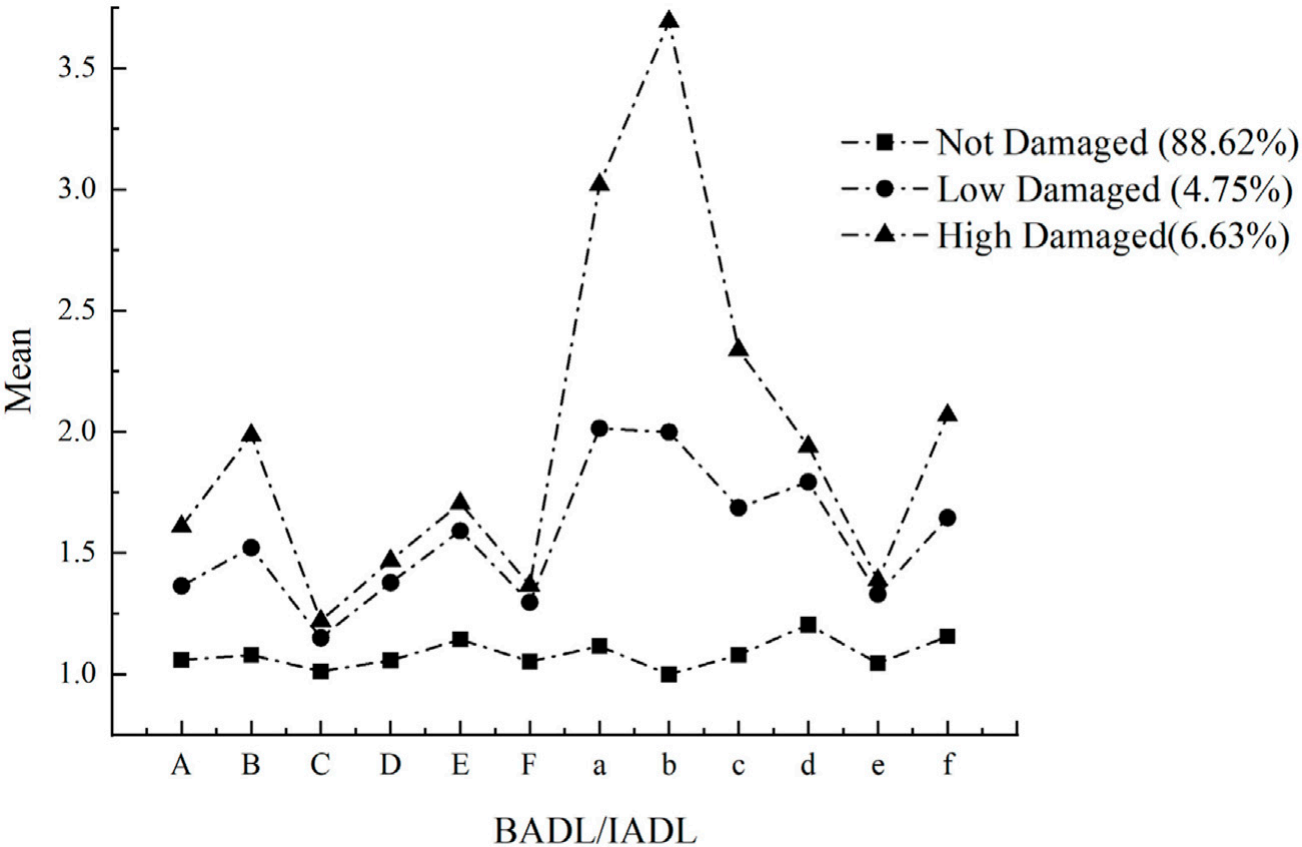
Parameters for the final 3-class patterns. Note. BADL: basic activities of daily living; IADL: instrumental activities of daily living. A: dressing; B: bathing; C: eating; D: getting in/out bed; E: using the toilet; F: controlling urination or defecation. a: doing housework; b: meal preparation; c: shopping; d: phoning; e: taking medication; f: managing money (China, 2024).

As shown in Table 1, one to four latent subgroups were checked based on fitting indicators. The results of LMR and BLRT were not significant for the 4-profile solution (*p* > 0.05), which indicated it was not appropriate. Compared to the 2-profile solution, the 3-profile solution performed better, showing a lower AIC, BIC, and aBIC, and the *p* values of both LMR and BLRT were significant. This indicated that the 3-profile solution offered the best available balance between parsimony and model fit. Profile labels were named based on classifications and illustrated in Figure 1. Three latent subgroups of ADL were identified: 88.62% were *Not Damaged* (no impairment of bodily function), 4.75% were *Low Damaged* (low impairment of bodily function), and 6.63% were *High Damaged* (high impairment of bodily function). Moreover, Table 2 summarizes the descriptive statistics for different ADL subgroups. The type of ADL is significantly correlated with age (*F =* 101.74, *p* < 0.001), gender (*χ* ^2^ *=* 80.48, *p* < 0.001), educational level (*χ* ^2^ *=* 32.95, *p* < 0.001), marital status (*χ* ^2^ *=* 53.32 *p* < 0.001) and household registration (*χ* ^2^ *=* 15.50, *p* < 0.001).

**TABLE 1:** Fitting index and group size of latent profile analysis models (China, 2024).

Model	AIC	BIC	aBIC	Entropy	LMR (*p*)	BLRT (*p*)
1	133,830.49	133,998.81	133,922.54	-	-	-
2	107,531.81	107,791.30	107,673.72	0.999	<0.001	<0.001
3	96,931.94	97,282.60	97,123.71	0.999	<0.001	<0.001
4	85,927.94	86,369.77	86,169.57	0.997	0.75	0.75

**TABLE 2:** Descriptive statistics for activity of daily living subgroups (China, 2024).

Variables	*Not Damaged* n = 7277	*Low Damaged* n = 390	*High Damaged* n = 544	Statistics (*p*)
Age, *M* (*SD*)	67.82 (5.99)	70.04 (6.58)	71.29 (7.17)	101.74 (<0.001)
Gender				
Women	3,473 (42.30)	117 (1.42)	310 (3.78)	80.48 (<0.001)
Men	3,804 (46.33)	273 (3.32)	234 (2.85)	
Education level				
Junior high school and below	6,433 (78.35)	373 (4.54)	510 (6.21)	32.95 (<0.001)
High school and above	844 (10.28)	17 (0.21)	34 (0.41)	
Marital status				
Divorced or widowed	1,194 (14.54)	116 (1.41)	118 (1.44)	53.32 (<0.001)
Married or cohabited	6,083 (74.08)	274 (3.34)	426 (5.19)	
Household registration				
Non-agricultural account	1,943 (23.66)	74 (0.90)	123 (1.50)	15.50 (<0.001)
Agricultural account	5,334 (64.86)	316 (3.85)	421 (5.13)	
ADL, *M* (*SD*)	13.12 (2.11)	18.73 (3.95)	23.87 (6.46)	

### Analysis of Variance Results

As shown in Table 3, based on the result of LPA, we found significant differences in life satisfaction (*F* = 331.00) and depressive symptoms (*F* = 56.19) across three subgroups of ADL. Moreover, multiple comparisons showed that older adults in the *Low Damaged* group have a lower level of life satisfaction than the other two groups. No significant difference in life satisfaction between the *High Damaged* and the *Not Damaged*. Compared to the *High Damaged*, the *Low Damaged* had more depressive symptoms. Compared to the *Not Damaged*, the *High Damaged* had more depressive symptoms.

**TABLE 3:** Analysis of variance results (China, 2024).

Variables	ADL profiles (*M* ± *SD*)			*F*	Multiple Comparisons
	*Not Damaged* 1	*Low Damaged* 2	*High Damaged* 3		
Life satisfaction	2.67 ± 0.75	3.01 ± 0.95	2.91 ± 0.93	331.00***	2 > 1; 2 > 3
Depressive symptoms	18.07 ± 6.31	24.50 ± 7.18	23.25 ± 7.30	56.19***	2 > 3 > 1

Note. ADL, activity of daily living; Level of confidence: 95%; ***p < 0.001.

### Mediating Effect of Life Satisfaction

**FIGURE 2:**
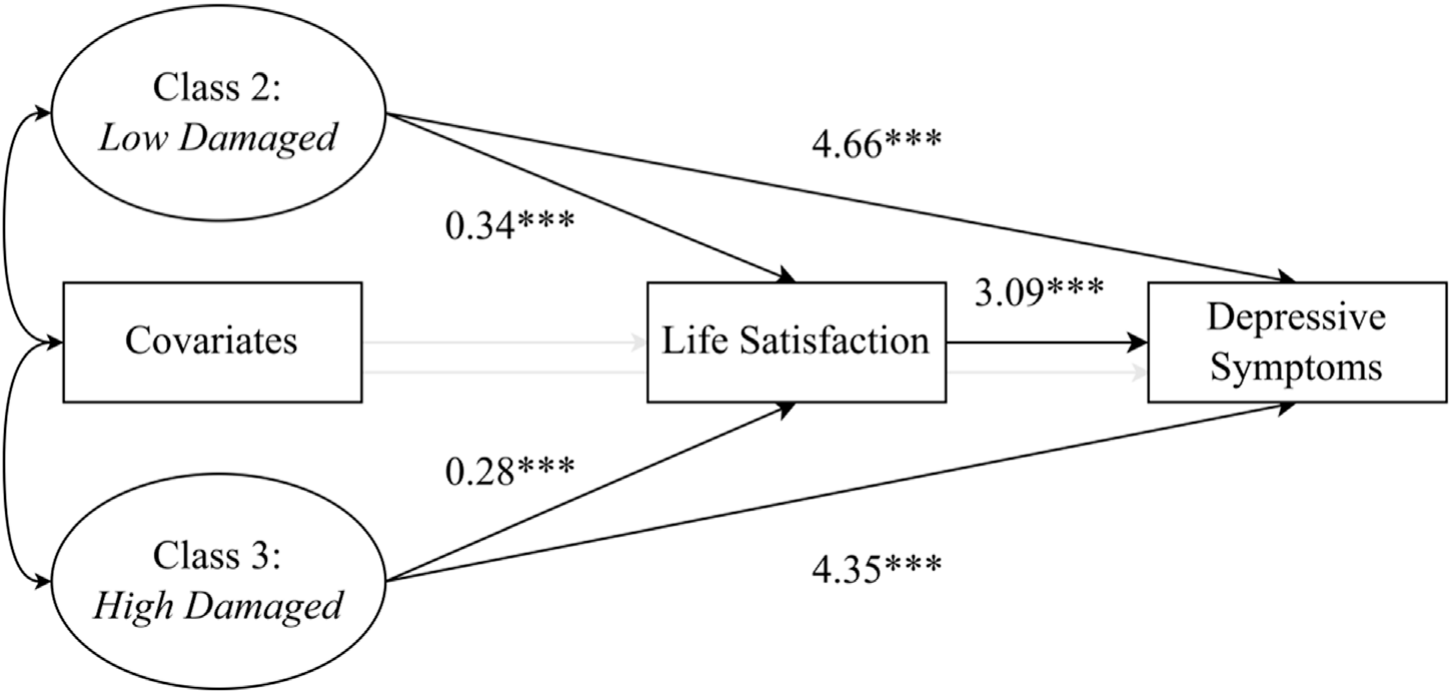
Model of the mediating role of life satisfaction. Note. Reference group: *Not Damaged*; Level of confidence: 95%; ***p < 0.001 (China, 2024).

All potential confounders were controlled in advance (Table 4; Figure 2). Taking *Not Damaged* as the reference group, the mediating effect size of *Low Damaged* on depressive symptoms through life satisfaction was 1.06 (95% CI: 0.76–1.36, *p* < 0.001), and the direct effect size was 4.66 (95% CI: 4.07–5.26, *p* < 0.001), and the mediation effect was accountable for 18.54%.; the mediating effect size of *High Damaged* on depressive symptoms through life satisfaction was 0.85 (95% CI: 0.61–1.11, *p* < 0.001), and the direct effect size was 4.35 (95% CI: 3.84–4.86, *p* < 0.001), and the mediation effect was accountable for 16.34%. Moreover, taking *Not Damaged* as the reference group, the coefficient of life satisfaction of *Low Damaged* was 0.34 higher than that of *Not Damaged*, so the level of depressive symptoms increased 3.09 accordingly; the coefficient of life satisfaction of *High Damaged* was 0.28 higher than that of *Not Damaged*, so the level of depressive symptoms increased 3.09 accordingly. Therefore, taking *Not Damaged* as the reference group, the relationship between other subgroups of ADL and depressive symptoms was partially mediated by life satisfaction.

**TABLE 4:** Testing for the mediation model (China, 2024).

Effect Decomposition	Path	Effect	*SE*	*LLCI*	*ULCI*
Total effect	*Low Damaged* → depressive symptoms	5.72***	0.33	5.08	6.37
Direct effect	*Low Damaged* → depressive symptoms	4.66***	0.30	4.07	5.26
Indirect effect	*Low Damaged* → life satisfaction → depressive symptoms	1.06***	0.15	0.76	1.36
Total effect	*High Damaged* → depressive symptoms	5.20***	0.28	4.65	5.75
Direct effect	*High Damaged* → depressive symptoms	4.35***	0.26	3.84	4.86
Indirect effect	*High Damaged* → life satisfaction → depressive symptoms	0.85***	0.13	0.61	1.11

Note. Reference group: *Not Damaged*; Level of confidence: 95%; ***p < 0.001.

## Discussion

This study examined scientific subgroups of ADL in older adults using a nationally-representative sample, exploring the link between different subgroups of ADL and depressive symptoms. Two vulnerable subgroups of ADL (*Low Damaged* and *High Damaged*) were identified, which had significantly lower life satisfaction and higher levels of depressive symptoms, along with another subgroup of ADL (*Not Damaged*). Using LPA to capture different ADL patterns helped identify heterogeneity in response patterns across various constructs, revealing the multidimensionality in ADL outcomes [21, 65]. Furthermore, life satisfaction was identified as a key mechanism linking ADL to depressive symptoms, especially in the vulnerable groups. Thus, targeted health services can be provided, ultimately reducing their depressive symptoms while enhancing life satisfaction for older adults [20, 24].

### Subgroups of ADL

By categorizing the ADL of older adults into potential groups, two vulnerable subgroups were identified (*Low Damaged* and *High Damaged*), along with another subgroup of ADL (*Not Damaged*). In the *Not Damaged* group, the older adults were not limited in their physical function, and the levels of BADL and IADL were extremely low. In the *Low Damaged* group, the physical function of the older adults was limited to a low degree, and the levels of BADL and IADL were relatively low. Finally, in the *High Damaged* group, the physical function of the older adults was limited to a high degree, and the levels of BADL and IADL were relatively high. Moreover, from a specific dimension, LPA clustering in the present study showed that IADL impairment was greater than BADL impairment in the *High Damaged* and *Low Damaged* groups. This is because the activities involved in IADL are significantly complex, diverse, and susceptible to external influences, whereas the activities involved in BADL are significantly basic, fixed, and autonomous [33, 66]. In contrast to the results of previous studies, our study found the types of impairment of both BADL and IADL but did not find the types of impairment of only one dimension while the other was unaffected [21]. Moreover, 88.62% of the participants were assigned almost physical functional independence (*Not Damaged*). In contrast, the proportion of almost physically functional independent individuals was lower in the study using the Chinese Longitudinal Healthy Longevity Survey data (74.77%) [21]. The difference in the results may be due to the different measurement tools used. Compared to previous classifications of ADL severity, although this study’s LPA results also categorize ADL into *Not Damaged, Low Damaged*, and *High Damaged*, our approach comprehensively considers the intrinsic characteristics of various dimensions. Consequently, the classification results have achieved higher accuracy, robustness, and scientific validity, thus providing more precise guidance for health interventions for the older adults [21, 62]. These findings highlight the need to respect individual differences and provide diverse care services based on the type and severity of ADL impairment, promoting successful aging and more effective assistance for older adults especially in China [67].

Moreover, descriptive statistical analysis revealed that older adults who were of advanced age, female, less educated, married or cohabiting, and registered in rural areas demonstrated relatively higher levels of impairment in ADL. This finding is consistent with previous studies and reflects entrenched health and social inequalities [12, 13, 17–19]. Advanced age naturally leads to chronic disease accumulation and physiological decline, making the oldest seniors more likely to experience ADL limitations. Likewise, older women tend to live longer than men but with greater morbidity. Low educational attainment is typically associated with a lifetime of lower socioeconomic status, poor health literacy, and physically demanding work. Rural elders often have less access to quality healthcare, lower income, and fewer support services, which exacerbates ADL dependence. Although a spouse may provide care and emotional support, aging couples may also share unhealthy lifestyles or caregiving burdens, potentially compounding each partner’s functional decline. Therefore, identifying vulnerable groups in terms of ADL based on socio-demographic factors is important for improving the health outcomes of older adults.

### Life Satisfaction and Depressive Symptoms in Three Subgroups

We discovered substantial variations in life satisfaction and depressive symptoms across the three ADL subgroups. Specifically, compared with the *Not Damaged* group, the level of life satisfaction was lower in the *Low Damaged* group, and the level of depressive symptoms was higher in the *Low Damaged* group and *High Damaged* group. These findings are consistent with previous study results [12, 13]. Physical dysfunction is a type of chronic stress [29, 30]. First, a decline in ADL is often accompanied by changes in cognitive function [36]. For older adults, there is a significant association between cognitive impairment and depressive symptoms, which affects their evaluation of the external environment and decreases their quality of life [33, 35]. Second, physical disabilities can affect the social participation of older people, leading to decreased social activities and a lack of interpersonal communication [34, 49]. Finally, limitations in physical function can cause psychological discomfort and panic among older adults, and dependence on others can result in loss of autonomy [32, 50]. These stress factors can significantly affect life satisfaction and depressive symptoms [31]. In contrast to previous studies, we found lower life satisfaction and higher levels of depressive symptoms in the *Low Damaged* group compared to the *High Damaged* group. Filial piety is the spiritual core of Chinese traditional culture, and adult children have an inescapable duty to support their parents [68]. Consequently, adult children offer financial assistance, life care, and spiritual consolation to older adults of the *High Damaged* group since they are unable to care for themselves. Such intergenerational supports may help relieve stress and lessen depressive symptoms [69]. A low degree of physical impairment impacts the quality of life of older adults in the *Low Damaged* group, which is inconvenient for their daily life [12]. However, older adult parents typically need the care of their adult children only when their physical condition is already very poor, as they do not want to place a burden on their adult children [50, 68]. Therefore, a double-pressure background can lead to a rapid increase in depressive symptoms in the *Low Damaged* group [70]. Our findings support the diathesis-stress model in explaining the relationship between ADL impairment, life satisfaction, and depressive symptoms in older adults. According to these findings, we suggest that adult children should prioritize the health of their older adult parents with impairments, particularly those with low levels of impairment within the family. This is because these older adult parents may be more prone to being overlooked or neglected, which may worsen their health condition.

### Mediating Role of Life Satisfaction

Using methods from previous studies [64, 65], we found that taking *Not Damaged* as the reference group led to the *Low Damaged* and *High Damaged* groups revealing low levels of life satisfaction. This is implicated in increasing depressive symptoms with the coefficient being relatively high in the *Low Damaged* group. According to the diathesis-stress model, chronic stress can accumulate and impact mental health [29, 30]. ADL reflects the chronic stress of daily life in older adults [31]. Compared with the older adults with physical dysfunction (*Low Damaged* and *High Damaged*), the older adults in the *Not Damaged* group can independently complete daily living activities, fostering confidence and autonomy, which enhances life satisfaction [48].

In contrast, those in the *Low Damaged* and *High Damaged* groups, with lower ADL levels, rely on others or assistive devices, leading to feelings of helplessness and loss, which diminish life satisfaction [49, 50]. Moreover, life satisfaction is an evaluation of needs and wishes for one’s own life status, and impacts the emotional wellbeing [38]. If older adults are content with their lives, they are likely to experience less stress, which can help reduce or prevent the onset of depressive symptoms [46, 47]. Conversely, older adults who are dissatisfied with their life are more likely to experience pressure, which raises the possibility that they may suffer from depressive symptoms [45]. Thus, life satisfaction mediates the relationship between ADL impairment and depressive symptoms, particularly in vulnerable subgroups. Our study provides a horizontal extension, suggesting that life satisfaction is a crucial transmission mechanism through which ADL affects depressive symptoms in older adults. Moreover, the results suggest that it may be possible to prevent older adults’ depressive symptoms, particularly in the vulnerable subgroups, with specific impairments in physical functioning, by improving their life satisfaction.

### Limitations and Implications

While this study offers valuable insights into the multidimensional aspects of ADL, its association with depressive symptoms, and the mediation mechanisms involved, it has some limitations. First, this study did not establish causal mechanisms between ADL and depressive symptoms, which future longitudinal studies should address. Second, ADL variables were self-reported rather than clinically diagnosed, potentially affecting measurement validity. Finally, this study focused on mediating mechanisms without exploring moderating factors, an area for future research to investigate.

Despite these limitations, the study makes significant contributions. Using a representative sample, it enhances our understanding of older adults’ health. Guided by the diathesis-stress model, the study identifies potential ADL subgroups and examines the mediating role of life satisfaction in the relationship between ADL subgroups and depressive symptoms. This approach provides a better understanding of the relationship between physical dysfunction and depressive symptoms in older adults. In terms of practical value, it is crucial to focus more on older adults with decreased physical function, particularly those in two vulnerable subgroups.

### Conclusion

In this study, using CHARLS data (wave 4), we focused on the relationship between ADL, life satisfaction, and depressive symptoms. There existed heterogeneity in the ADL of Chinese older adults, and three latent subgroups were identified: *Not Damaged*, *Low Damaged*, and *High Damaged*. The vulnerable subgroups of ADL (*Low Damaged* and *High Damaged*) had lower life satisfaction and high levels of depressive symptoms. Moreover, taking *Not Damaged* as the reference group, the relationship between other subgroups and depressive symptoms was partially mediated by life satisfaction. The results of this study may help the older adults in vulnerable subgroups with different physical disorders to improve their health, so as to effectively cope with the problem of population aging.
